# Analysis of the differential urinary protein profile in IgA nephropathy patients of Uygur ethnicity

**DOI:** 10.1186/s12882-018-1139-3

**Published:** 2018-12-14

**Authors:** Zhengguang Guo, Zhao Wang, Chen Lu, Shufen Yang, Haidan Sun, Reziw, Yu Guo, Wei Sun, Hua Yue

**Affiliations:** 1 Core Facility of Instrument, Institute of Basic Medicine, Chinese Academy of Medical Sciences, School of Basic Medicine, Peking Union Medical College, 5 Dong Dan San Tiao, Beijing, China; 2 Nephrology department, The Xinjiang Uygur Autonomous Region People’s Hospital, 91 Tianchi Road, Urumqi, Xinjiang China; 3 Graduate School, Xinjiang Medical University, 393 Xinyi Road, Urumqi, Xinjiang China

**Keywords:** IgA nephropathy, Urinary proteomics, Uygur, Biomarker

## Abstract

**Background:**

IgA nephropathy (IgAN) is one of the most common forms of idiopathic glomerular diseases and might lead to end-stage kidney disease. Accurate and non-invasive biomarkers for early diagnosis are required for early intervention and consequent therapy for IgAN patients. Because variance in the disease incidence and predisposing genes of IgAN has been detected among different ethnicities, the ethnicity factor should be considered in IgAN biomarker discovery. The differences in the protein profiles and pathological mechanisms of IgAN in patients of Uygur ethnicity need to be clearly illustrated.

**Methods:**

In this study, we used urinary proteomics to discover candidate biomarkers of IgAN in patients of Uygur ethnicity. The urinary proteins from Uygur normal control and Uygur IgAN patients were extracted and analyzed using 2D-LC-MS/MS and isobaric tags for relative and absolute quantitation (iTRAQ) analysis.

**Results:**

A total of 277 proteins were found to be differentially represented in Uygur IgAN compared with the respective normal controls. The bioinformatics analysis revealed that the immune response, cell survival, and complement system were activated in Uygur IgAN. Many differentially expressed proteins were found to be related to nephropathy and kidney injuries. Four candidate biomarkers were validated by Western blot, and these results were consistent with the iTRAQ results. ICAM1, TIMP1, SERPINC1 and ADIPOQ were upregulated in Uygur IgAN. Bioinformatic analysis revealed that the increase of ICAM1 and TIMP1 might be caused by IgAN, but the increase of SERPINC1 and ADIPOQ might be caused by proteinuria. SERPINC1 and ICAM1 were identified as the candidate biomarkers with excellent area-under-the-curve (AUC) values (0.84) for distinguishing Uygur IgAN from normal controls.

**Conclusions:**

Using urinary proteomic analysis, we identified several candidate biomarkers for IgAN in patients of Uygur ethnicity. These results will prove helpful for exploring the pathological mechanism of IgAN in patients of Uygur ethnicity and for developing better treatments for these patients.

**Electronic supplementary material:**

The online version of this article (10.1186/s12882-018-1139-3) contains supplementary material, which is available to authorized users.

## Introduction

IgA nephropathy (IgAN) is one of the most common forms of idiopathic glomerular diseases [1], and primary IgAN is characterized by the deposition of the IgA antibody in the glomerulus [2]. Overall, 25–30% of all cases of IgAN lead to end-stage disease over a period of 20 years [3]. Early intervention and consequent therapy could prevent or delay the development of renal failure and will reduce the number of patients who require renal replacement therapy [4]. The diagnosis of IgAN requires a renal biopsy, which it is an invasive procedure with a low risk of serious bleeding complications [5]. Therefore, accurate and non-invasive biomarkers for the early diagnosis of IgAN are required.

The urinary proteome can reflect alterations in the urinary system; thus, urine is a suitable source for the discovery of biomarkers of kidney diseases [6]. As early as 2006, Mi-Ra Park et al. used 2-DE and MALDI-TOF MS to identify the urinary proteins showing differential expression in IgAN and normal controls and determined that 59 proteins were differentially expressed in IgAN [7]. To date, many publications have reported differentially expressed proteins in the urine, urinary exosomes and kidney tissue of IgAN patients using various proteomic methods, including MALDI-TOF [4, 7, 8], 2D-GEL [1], iTRAQ [9, 10], SILAC [11], and label-free quantification methods [12, 13]. A total of 166 candidate biomarkers have been discovered, and some of these have been validated through ELISA or Western blot analysis. The above-mentioned studies suggest that urinary proteomics could be used for the discovery of biomarkers of IgAN, and more accurate candidate urinary biomarkers for IgAN must be identified and validated.

Although IgAN has been detected among all ethnicities worldwide, it displays striking variations among different ethnicities. Specifically, IgAN is most common in Asians, moderately prevalent in Europeans, and rare in Africans [14]. Krzysztof et al. recently performed a genome-wide association study (GWAS) to identify and confirm that genome variances across a multi-ethnic cohort contribute to disease risk in different ethnicities [14]. Therefore, the ethnicity factor should be considered in the discovery of urinary biomarkers for IgAN.

Uygur is an important minority in the Xinjiang District of China, which contains over 10 million people, and the incidence of IgAN in the Uygur population is 12.5% [15, 16], which is higher than common chinese population (5%, ranged from 3 to 9%) [17–20]. In our previous study, which was performed at the genome level, we identified five SNPs of C1GalT1 (b1,3-galactosyltransferase) [15] and T cell receptor alpha chain constant gene (TCRC alpha)-560 C/T polymorphism in the Uygur ethnicity [21] and assessed the relationship between genotype and the clinical presentation of IgAN in the Uygur cohort. However, the urinary proteomics of IgAN in patients of Uygur ethnicity has not been previously investigated.

**Fig. 1:**
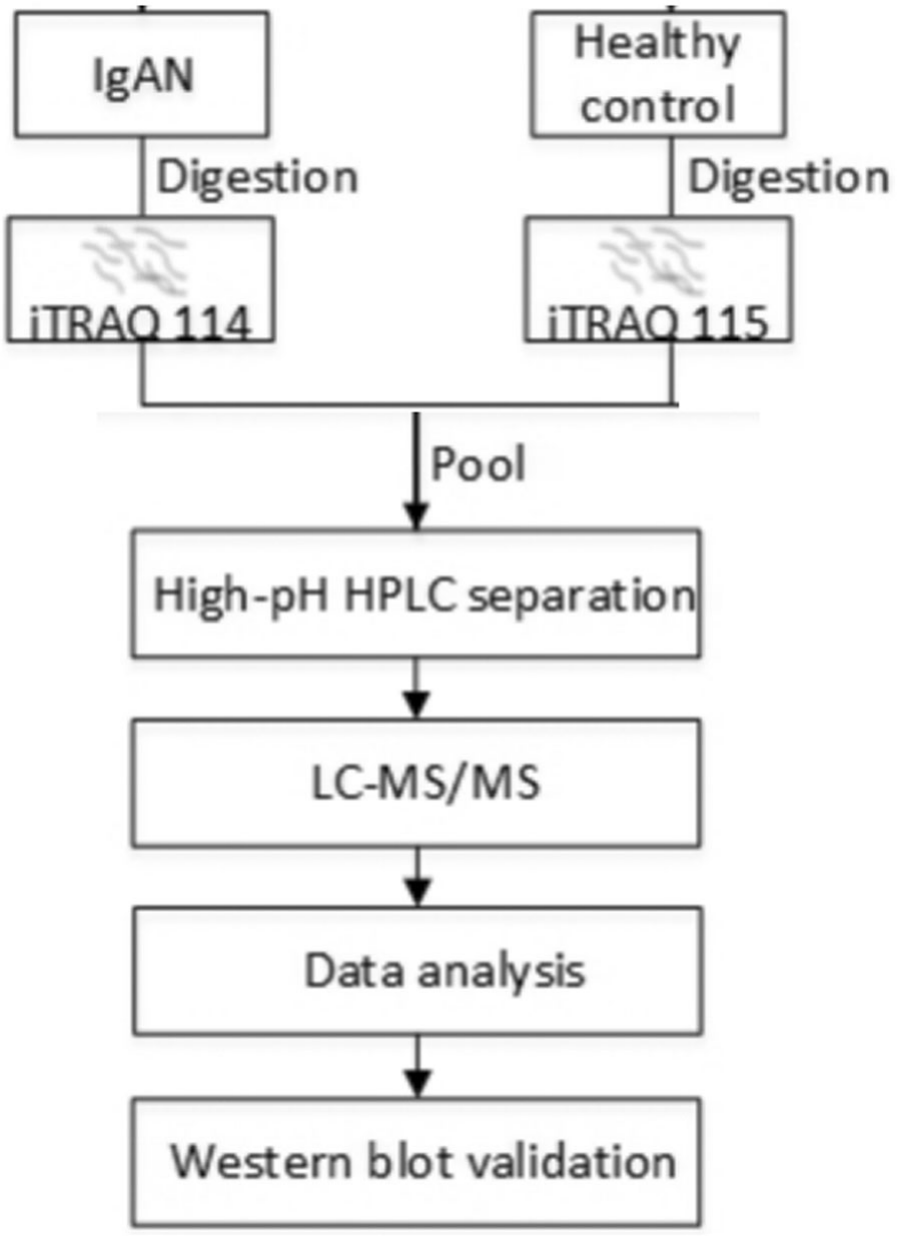
Work flow of the analysis of the differential urinary proteome in Uygur IgAN

In this study, the urinary proteome was investigated to discover candidate biomarkers for IgAN in the Uygur ethnicity. Urinary proteins were collected from Uygur normal controls and Uygur IgAN patients, and these two groups of pooled proteins were labeled with four-plex isobaric tags for relative and absolute quantitation (iTRAQ) reagents and analyzed by 2D-LC MS/MS. The proteins showing differential expression between IgAN patients and normal controls were functionally analyzed through Panther GO and Ingenuity Pathway Analysis (IPA). Furthermore, some select differentially expressed proteins were validated in individual samples by Western blot (Fig. 1).

## Materials and methods

### Reagents and instruments

HPLC-grade acetonitrile (ACN), formic acid, dithiothreitol (DTT) and iodoacetamide (IAM) were purchased from Sigma (St. Louis, MO, USA). Sequencing-grade trypsin was purchased from Promega (Madison, WI, USA). The four-plex iTRAQ reagents were purchased from ABsciex (Framingham, MA, USA). A TripleTOF 5600 mass spectrometer from ABsciex and an HPLC system from Waters (Milford, MA, USA) were used.

For the Western blot analysis, primary antibodies for adiponectin (ADIPOQ, HPA051767), intercellular adhesion molecule 1 (ICAM1, HPA002126) and antithrombin-III (SERPINC1, HPA001816) were purchased from Sigma, and primary antibodies for metalloproteinase inhibitor 1 (TIMP1, ab109125) were purchased from Abcam (Cambridge, UK).

### Patient selection

In this study, 24 participants, consisting of 12 Uygur IgAN patients and 12 Uygur healthy controls, were recruited. Each group was divided into an experimental group (*n* = 5) for proteomic analysis and a validation group (*n* = 7) for Western blot validation. The clinical characteristics of all of the participants are shown in Table 1 (detailed information is provided in Additional file 1: Table S1). The age, systolic blood pressure (SBP), diastolic blood pressure (DBP), body mass index (BMI), serum creatinine (SCr), blood urea nitrogen (BUN), urinary albumin excretion rates (UAER), estimated glomerular filtration rate (eGFR), triglycerides (TG), serum albumin (Alb), low-density lipoprotein (LDL), high-density lipoprotein (HDL) and fasting blood glucose (FBG) in the two groups were measured. TThis study was approved by the Institutional Review Board of the Institute of Basic Medical Sciences and conformed to the principles outlined in the Declaration of Helsinki. Committee’s reference number: 036–2014. Because our study used the remaining waste samples of the participants from clinical examination, and did not directly contact with the participants and would not regarded privacy of the participants, the Institutional Review Board of the Institute of Basic Medical Sciences waived the informed consents.

**Table 1:** Clinical characteristic of Uygur normal controls and IgAN patients

Characteristics	Uygur normal controls (*n* = 12)	Uygur IgAN patients (*n* = 12)
Gender (M/F)	9/3	6/6
Age (year)	43.8 ± 8.1	38.7 ± 8.3
BMI (mg/m2)	21.1 ± 1.7	24.3 ± 2.8^b^
SCr (μmol/L)	86.9 ± 9.7	166.2 ± 182.1
BUN (mmol/L)	5.9 ± 1.2	9.1 ± 5.9^a^
UAE (μg/min)	11.8 ± 1.8	349.8 ± 115.1^b^
eGFR (ml/min)	97.9 ± 7	65.4 ± 34.2^b^
Alb (g/l)	48.9 ± 4.5	38.6 ± 4.9^b^
TG (mmol/L)	1.3 ± 0.4	1.7 ± 0.9
SBP (mmHg)	128.1 ± 10.3	132.7 ± 17.9
DBP (mmHg)	80.2 ± 5.3	83.3 ± 11.1
LDL (mmol/L)	2.6 ± 0.4	2.7 ± 0.9
HDL (mmol/L)	1.5 ± 0.4	1.2 ± 0.3
FBG (mmol/L)	4.6 ± 0.3	4.5 ± 0.8

*BMI* body mass index, *SCr* serum creatinine, *BUN* blood urea nitrogen, *UAE* urine albumin, *eGFR* estimated glomerular filtration rate, *Alb* serum albumin, *TG* triglyceride, *SBP* systolic blood pressure, *DBP* diastolic blood pressure, *LDL* low-density lipoprotein, *HDL* high-density lipoprotein, *FBG* fasting blood glucose

^a^*p* < 0.05 for IgAN versus its respective normal control; ^b^*p* < 0.001 for IgAN versus its respective normal control

### Urinary protein extraction, protein digestion, and iTRAQ labeling

The urinary protein extraction, protein digestion and iTRAQ labeling protocols used in this study were described in our previous publication [6]. In detail, urinary samples collected in the morning from the two groups were centrifuged at 5000 g for 30 min to remove the precipitates. The supernatants were precipitated by three volumes of ethanol and re-suspended in lysis buffer (containing 7 M urea, 2 M thiourea, 0.1 M DTT and 50 mM Tris). The protein concentration of each sample was estimated using the Bradford method. Equal amounts of normalized total protein were taken from each of the donors in the experimental groups (*n* = 5 in each group), and 200 μg of protein from each sample within each group was pooled.

The filter-aided sample preparation (FASP) method was used for protein digestion with trypsin. First, 1 mg of the pooled proteins in the control and patient groups was reduced by 20 mM DTT at 37 °C for 1 h and alkylated by 50 mM IAM at room temperature in the dark for 45 min. The samples were then loaded onto a 10-kDa ultrafilter tube (Pall, Port Washington, NY, USA) and washed twice with 8 M urea and then twice with 25 mM NH_4_HCO_3_. After trypsin dissolved in 25 mM NH_4_HCO_3_ was added to the protein samples, the samples were digested at 37 °C overnight. The digested peptides were collected by filtration and then subjected to desalting using an Oasis C18 column. The 100-μg peptide in the IgAN and control samples was individually labeled with the 114 and 115 four-plex iTRAQ reagents according to the manufacturer’s protocol (ABsciex). We used equal amounts of total protein for sample normalization. Finally, the samples were pooled for 2D-LC-MS/MS analysis.

### 2D LC-MS/MS analysis

The pooled iTRAQ labeled samples were first fractionated using a high-pH RPLC column from Waters (4.6 mm × 250 mm, Xbridge C18, 3 μm). A mass of 200 μg of the pooled iTRAQ-labeled samples was loaded onto the column in buffer A1 (0.1% aqueous ammonia in water, pH 10) and eluted with buffer B1 (0.1% aqueous ammonia in 10% water and 90% ACN, pH 10, flow rate = 0.6 mL/min) with a gradient ranging from 5 to 25% for 60 min. The eluted peptides were collected as 60 fractions, with one fraction collected per minute, and were pooled to obtain 20 samples. Each fraction was analyzed with a reverse-phase-C18 self-packed capillary LC column (75 μm × 100 mm). A TripleTOF 5600 mass spectrometer was used for the LC-MS analysis. The MS data were acquired under the conditions described in our previous publication [6]. Each fraction was run twice.

### Data processing

Mascot software (Matrix Science, London, UK; version 2.5.01) was used for the database searching of all of the samples. In Mascot, the database was set to the Swiss-Prot human database, and the digestion enzyme was set to trypsin. The parent ion mass tolerance was 0.02 Da, and the fragment ion was 0.05 Da. Carbamidomethyl of cysteine and the four-plex iTRAQ labeling were set as fixed modifications, and a maximum of two miscleavage sites was allowed. For protein identification, Scaffold (version Scaffold_4.0.7, Proteome Software Inc., Portland, OR, USA) was used. During the process of protein assembly, some shared peptides were assigned to a group of proteins but could not be assigned to a specific protein. These groups of proteins that were identified based on these shared peptides were clustered as a protein group. To acquire the confidential identification results, peptide and protein identification was set to a FDR of less than 1.0% on both the peptide and protein levels, and a protein group with at least one unique peptide that was not shared with other protein groups was set as the filtering criteria. To meet the principle of simplicity, Scaffold Q+ (version Scaffold_4.3.2, Proteome Software Inc., Portland, OR, USA) was used for the iTRAQ quantification. The acquired intensities in the experiment were normalized globally in all runs. The healthy controls were set as the reference channels, which were normalized to produce a 1:1-fold change, resulting in all of the proteins from the healthy controls having a value of 1. The reporter ion peak intensity below 1% of the highest peak intensity in a spectrum was unreliable for quantification and removed from the quantification [6].

To estimate the relative abundance of proteins in the urine of IgAN, the acquired .wiff files were imported into Progenesis software (Nonlinear Dynamics, Version 4.1) for label-free quantification analysis. The features were filtered for charge state (+ 2 to + 5). The MS/MS spectra from all runs were exported and searched against the SwissProt human database using Mascot with the same parameters above. The results were imported back into Progenesis, and the peptide identifications were mapped onto the quantitative peptide data. The protein abundance was summarized from all quantification from the 20 runs. The relative abundance of one protein is calculated by the abundance of one protein/ the abundance of all proteins in urine.

### GO and IPA analysis

For the GO analysis, all of the differential proteins were analyzed using the Panther database (http://www.pantherdb.org/) and compared with urinary database. The proteins were classified based on their molecular function, biological process and cellular component categories in the Gene Ontology (GO) annotations.

For the IPA analysis, the differential proteins were analyzed using IPA software (Ingenuity Systems, Mountain View, CA, USA). The proteins were mapped to the IPA database and other databases in the disease and the functional categories and canonical pathways categories with Z-score and *P*-value rankings, respectively.

### Western blot analysis

Four selected candidate biomarkers, namely ADIPOQ, SERPINC1, ICAM1, and TIMP1, were validated in individual samples from the experimental and validation groups by Western blot. In detail, the protein lysates were separated on a 6% SDS-polyacrylamide gel, electrotransferred to polyvinylidene fluoride (PVDF, Immobilon P, Millipore) membranes, and blocked using 5% nonfat dry milk in Tris-buffered saline at pH 7.5 (TBST, 100 mmol/L NaCl, 50 mmol/L Tris, and 0.1% Tween-20). The membranes were immunoblotted with primary antibodies against the candidate proteins and then with secondary antibodies that were conjugated to horseradish peroxidase (HRP) [6]. The chemiluminescence signals were collected using an LAS 4000 system (ImageQuant LAS 4000 mini, General Electric Company, Boston, MA, USA), and the subsequent quantitative analysis was performed using ImageJ software. The ROC curves were drawn using SPSS 18.0.

## Results

### Qualitative and quantitative analysis of the urinary proteome

IgAN patients and normal controls were included in this study. The UAER, SCr and BUN levels were increased by 28-fold, 91 and 54%, respectively, and the eGFR and serum Alb levels were decreased significantly by 33 and 21%, respectively, in the Uygur IgAN group compared with the Uygur normal controls. Additionally, the BMI index was increased in the Uygur IgAN patients. Other indexes showed no significant differences between the two groups (Table 1, detailed data are shown in Additional file 1: Table S1).

The urinary proteins from the normal control experimental group (*n* = 5) and the IgAN experimental group (*n* = 5) were extracted, and the samples from each group were pooled. Each of the pooled samples was subjected to iTRAQ labeling and analyzed by 2DLC-MS/MS. A search of the human Swiss-Prot database using the Mascot algorithm with a 1% false discovery rate (FDR) at both the peptide and protein levels identified a total of 1025 protein groups with at least one peptide. Specifically, a total of 911/877 proteins and 2724/2697 peptides were identified in the first/second run of the fractions (detailed data are shown in Additional file 2: Table S2). Using a ratio-fold change greater than 2, 277 proteins were found to be differentially represented in Uygur IgAN patients compared with the normal controls, and of these 233 and 44 were upregulated and downregulated, respectively (detailed data are shown in Additional file 3: Table S3).

### GO and IPA analyses

To further study the biological function of the proteins showing differential expression between Uygur IgAN patients and the normal controls, the 277 differentially expressed proteins were subjected to GO and IPA analyses.

**Fig. 2:**
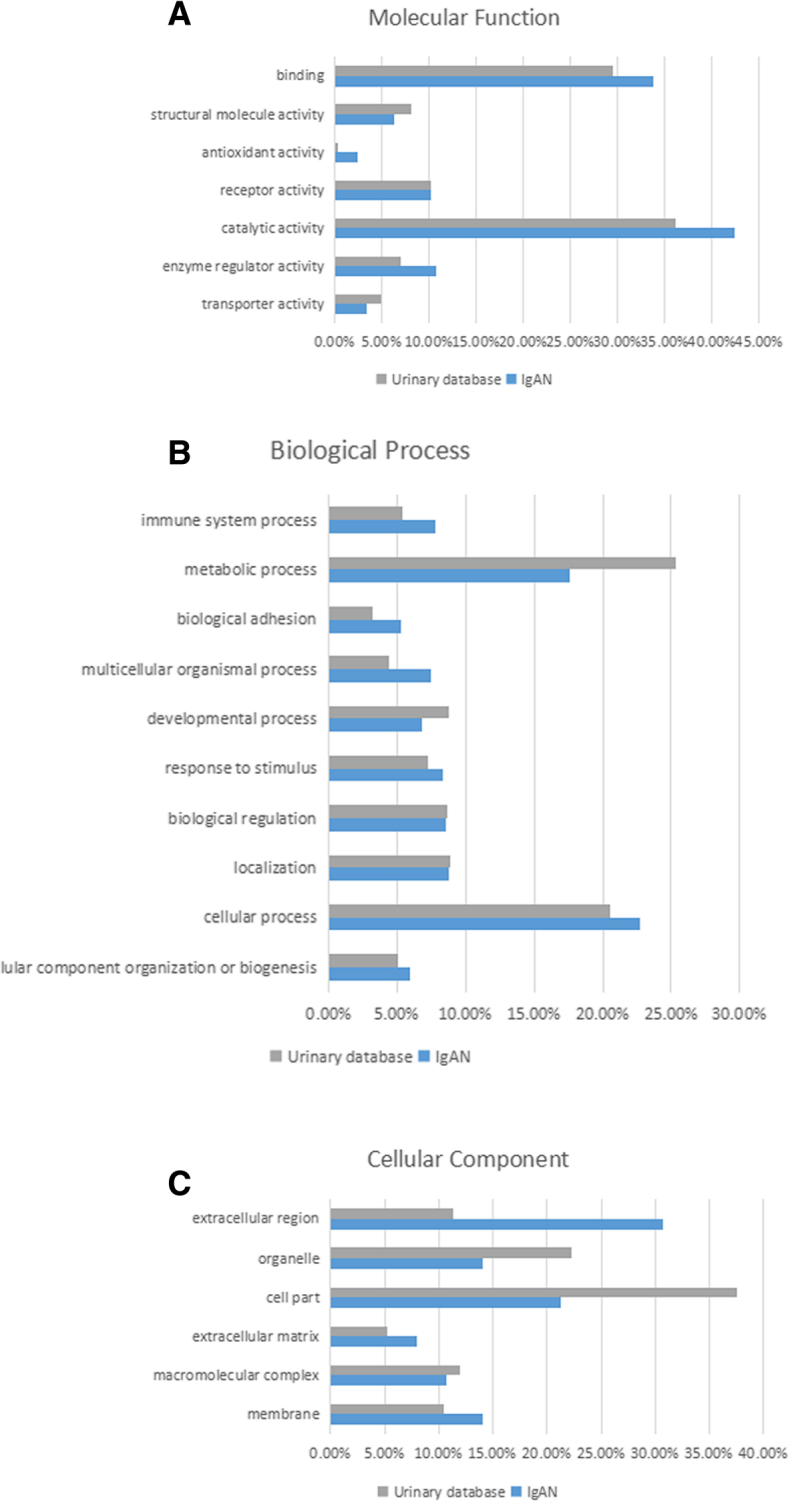
GO analysis of differentially expressed proteins in Uygur IgAN. Differentially expressed proteins in Uygur IgAN were classified into the molecular function (**a**), biological process (**b**), and cellular component (**c**) categories of human genes compared with the entire human normal urinary proteome through GO analysis. Categories with a constitution of at least 2% are displayed in the bar charts

The proteins showing differential expression in Uygur IgAN were searched for the enrichment of GO terms in the Panther Classification System with the urinary database. The differentially expressed proteins were classified into the molecular function, biological process and cellular component categorizes (Fig. 2). In the molecular function category, antioxidant activity was overrepresented in IgAN (*p*-value = 1.0E-08, enrichment score = 16). In the biological process category, the immune response process were overrepresented (*p*-value = 5.0E-11, enrichment score = 3.7). In the cellular component category, extracellular proteins (*p*-value = 3.2E-43, enrichment score = 3.6) and extracellular matrix proteins (*p*-value = 1.7E-04, enrichment score = 2.4) were overrepresented, whereas intracellular proteins were underrepresented in IgAN. Several extracellular matrix-related proteins, including MMPs and TIMPs, showed differential expression in the urine of IgAN patients, indicating that the deposition of extracellular proteins was increased in the mesangium in IgAN.

**Fig. 3:**
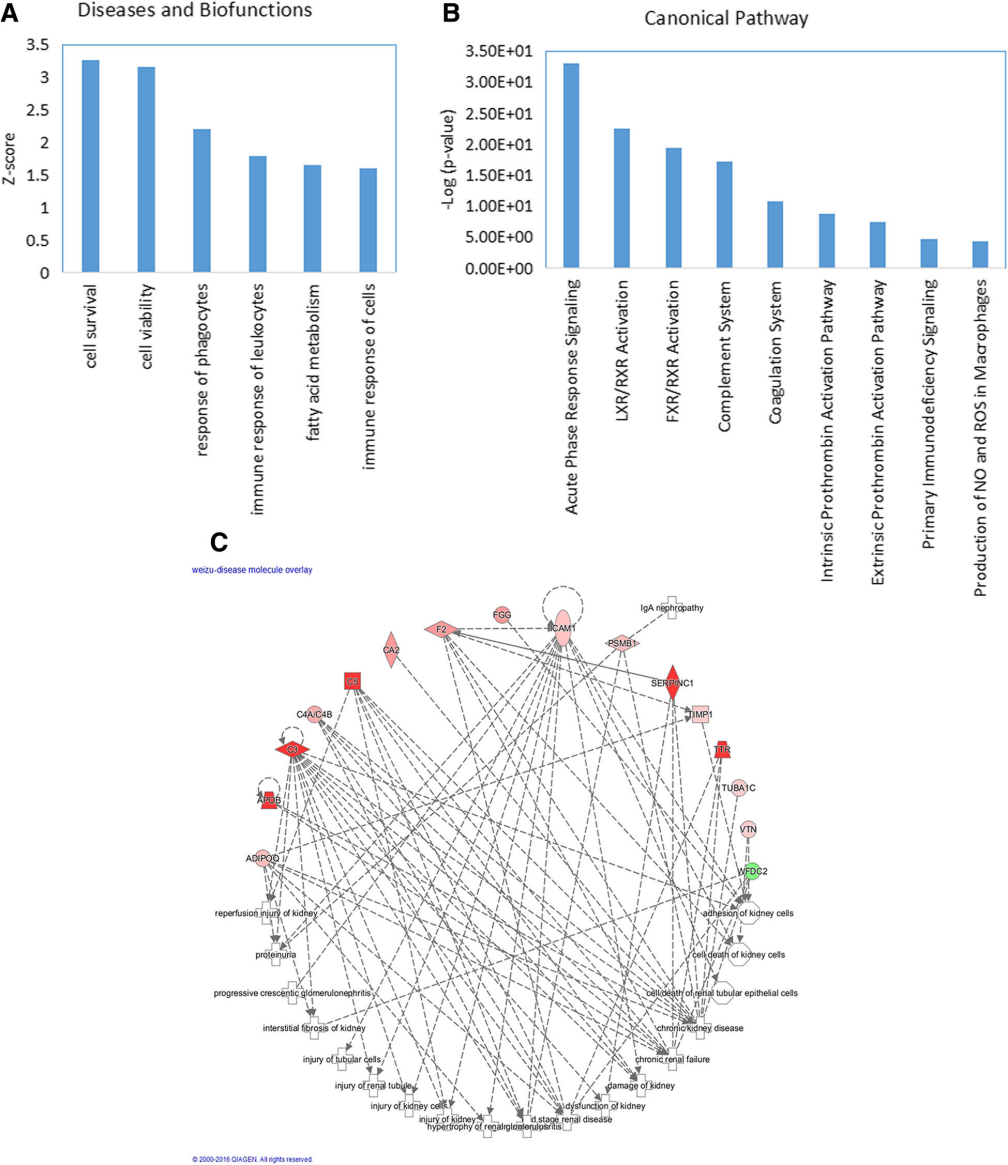
IPA analysis of differentially expressed proteins in Uygur IgAN**. a** Disease and biofunction analysis of differentially expressed proteins in Uygur IgAN. Z-score > 2, significantly activated; Z-score < − 2, significantly inhibited. **b** Top enriched canonical pathways in the Uygur IgAN group. -Log(*p*-value) > 1.5, significantly enriched. **c** Network showing that several important differentially expressed proteins in Uygur IgA nephropathy are associated with kidney dysfunction and disease

To further analyze the detailed functional changes in Uygur IgAN, an IPA analysis was performed. The disease and biofunction analysis revealed that the immune response and fatty acid metabolism were activated in IgAN. Additionally, cell viability and survival were activated in IgAN, which might reflect the compensatory response of the kidney to resist kidney damage caused by IgA nephropathy (Fig. 3a).

To detail the detection of the molecular mechanism of IgAN, a canonical pathway analysis was performed. For example, the immune response related pathway were activated in IgAN, which indicates that the immune system is activated in IgA nephropathy [22, 23]. The complement system was also found to be activated in Uygur IgAN, which demonstrates that local complement activation plays a critical role in glomerular injury in IgAN [22] By IPA analysis, we also found that 16 differential proteins associated with nephropathies or kidney dysfunctions. These proteins might be candidate biomarkers of IgAN (Fig. 3c).

Therefore, the urinary proteome could provide comprehensive pathological and physiological information of IgAN in patients of Uygur ethnicity.

### Western blot validation

By IPA analysis, four proteins, including ICAM1, TIMP1, ADIPOQ and SERPINC1 were functional related to IgAN or kidney injury in previous studies. SERPINC1 is associated with dysfunction of the kidney and chronic renal failure [10, 24]. ADIPOQ is associated with chronic kidney failure, end-stage renal disease, and renal hypertrophy [25–27]. TIMP1 play a role in the adhesion of kidney cells [28, 29]. ICAM1 is associated with proteinuria, renal tubule injury and kidney injury [30–34]. In addition, ADIPOQ and SERPINC1 have reported to be upregulated in the urine and plasma of IgAN in previous studies [10, 24, 35]. These four proteins were selected for western blot validation. (The evidences of the four proteins on IgAN were shown in Table 2). All the four proteins were validated in both the experimental group (*n* = 5) and the validation group (*n* = 7) of the IgAN patients (total *n* = 12) and normal controls (total *n* = 12).

**Table 2:** The evidence of the four differential proteins on IgAN or kidney injury in tissue level, body fluid level and functional level

Protein name	Kidney tissue evidence on IgAN	Kidney tissue evidence on IgAN method	Body floid evidence on IgAN	Body floid evidence on IgAN method	Evidence in kidney injury and other kidney diease
ADIPOQ			serum ↑ urinary↑ [35]	radio-immunity analysis	1. an adipocyte-specific plasma protein 2. accumulates in the injured kidney, modulating inflammation and oxidative stress. [25, 26] 3. induces kidney apoptosis in Ischemia-reperfusion injury [27].
SERPINC1	capillary walls of the glomeruli [24]	immunofluorescence	urinary↑ [10, 24]	Laurell rocket immunoelectrophoresis; iTRAQ quantification	1. Serine protease inhibitors in plasma that inhibits the blood coagulation cascade.
ICAM1	The tubular and interstitial expression [30]	avidin-biotin-peroxidase			1. a member of ligands for the leukocyte adhesion protein LFA-1 (integrin alpha-L/beta-2). 2. Increases in acute renal allograft rejection [31], chronic kidney disease (CKD) [32] and lupus nephritis [33, 34] in urine.
TIMP1					1. A positive regulator of ECMs in the mesangium. 2. Elevated in acute kidney injury (AKI) after liver transplantation [28] 3. Overexpressed in the kidney tissue of diabetic nephropathy rats [29].

**Fig. 4:**
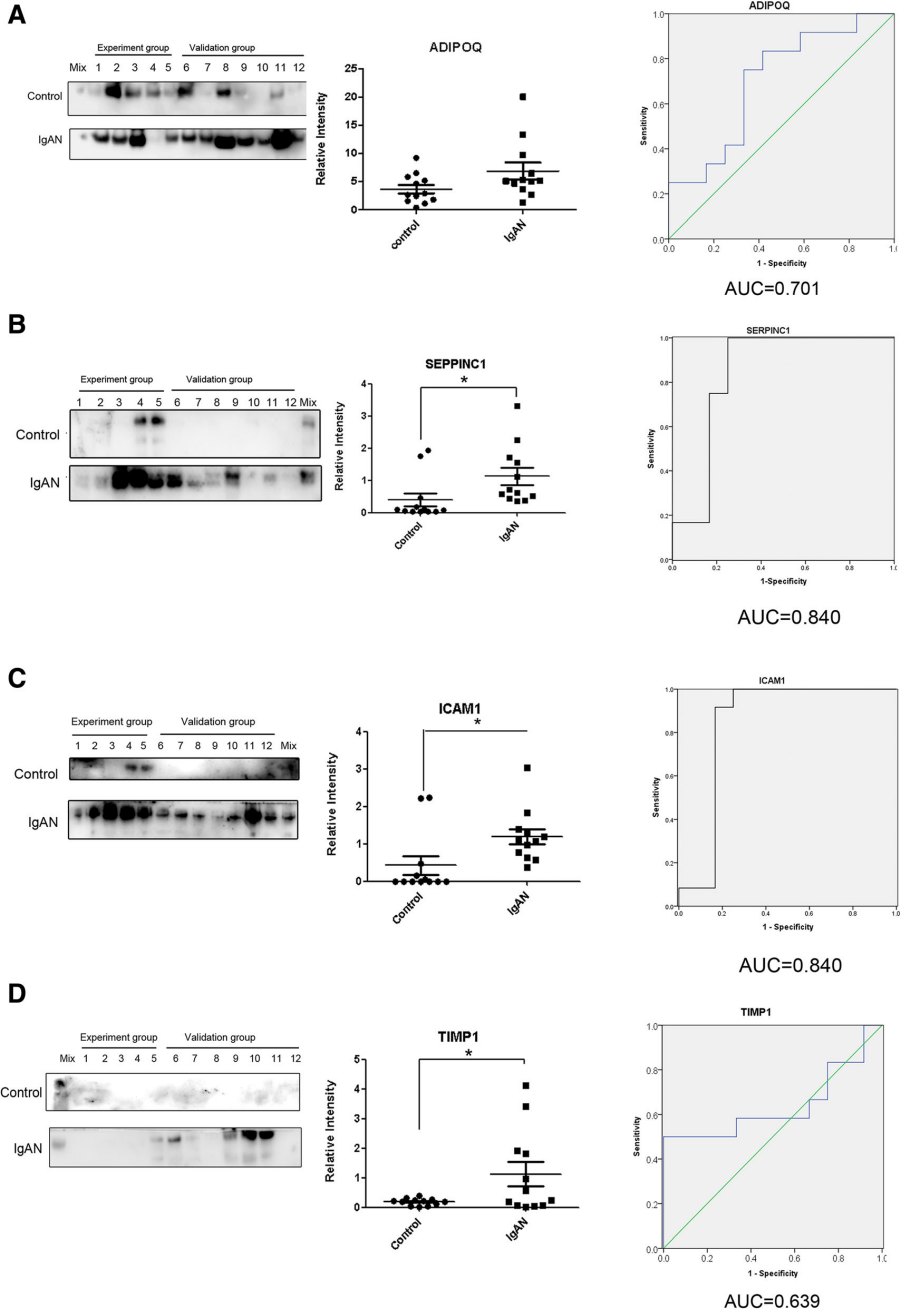
Western blot validation of four differentially expressed proteins. ADIPOQ (**a**), SERPINC1 (**b**), ICAM1 (**c**), and TIMP1 (**d**). * *p* < 0.05; . ROC curves for Western blot validation of ADIPOQ (**a**), SERPINC1 (**b**), ICAM1 (**c**) and TIMP1 (**d**) in the IgAN group versus the control group are shown

As shown in Table 3, the Western blot results for all four differentially expressed proteins revealed similar trends to those found through the iTRAQ analysis. SERPINC1, ADIPOQ, ICAM1, and TIMP1 were all upregulated in the Uygur IgAN patients (Fig 4a-d and Table 3).

**Table 3:** Quantitative results from the iTRAQ and Western blot analysis of four differentially expressed urinary proteins

Gene Name	Accession number	iTRAQ quantification	Western blot	AUC value
Adiponectin (ADIPOQ)	Q15848	1:3.94	1:1.87	0.701
Antithrombin-III (SERPINC1)	P01008	1:13.86	1:2.82^*^	0.840
Intercellular adhesion molecule 1 (ICAM1)	P05362	1: 2.06	1:2.77^*^	0.840
Metalloproteinase inhibitor 1 (TIMP1)	P01033	1:3.25	1:5.89^*^	0.639

(Normal control: IgAN). The AUC values of the ROCs of the four candidate biomarkers are shown

^*^p < 0.05

To evaluate the diagnostic effects of the four candidate biomarkers, which were found to be upregulated in Uygur IgAN, the ROC curves were plotted. As shown in Fig. 4a and d, ADIPOQ and TIMP1 showed moderate sensitivity and specificity for the diagnosis of IgAN, with area-under-the-curve (AUC) values of 0.701 and 0.639, respectively. In contrast, SERPINC1 (Fig. 4b) and ICAM1 (Fig. 4c) showed excellent sensitivity and specificity for the diagnosis of IgAN, with AUC values of 0.840 and 0.840, respectively, indicating that SERPINC1 and ICAM1 are potential biomarkers for the diagnosis of IgAN in the Uygur ethnicity. In addition, missing protein bands for the four proteins were observed in some cases, particularly in the normal control group, and this finding might be due to the fact that the amounts of the four proteins in some normal control cases were lower than the limit of detection by Western blot. This low amount of the four proteins in some cases might be caused by inter-individual variations. Previous studies have shown inter-individual variations in urinary proteins of 20–60% in normal humans [36, 37], and higher inter-individual variations might exist in IgAN patients.

## Discussion

In this study, we used a normal Uygur control group and IgAN patients of Uygur ethnicity to evaluate the characteristics of IgAN urinary proteomics in a Uygur cohort. A total of 277 proteins showed differential expression in the Uygur IgAN samples. By IPA analysis, we found that some of the differential proteins in IgAN like lipid and coagulation proteins were found abundantly in serum and thus likely serum-leaked into urine. To detect whether the four IgAN potential biomarkers were leaked from serum or not, we compared the relative abundance of the four proteins in urine of IgAN, and in the serum (data from Geyer’s study [38]). The serum protein data were generated from quantitative label-free LC-MS/MS analysis of normal human serum and the protein abundance were evaluated using peptide intensity [38]. We also analyzed the IgAN differential protein abundance in urine using the same method. If the relative abundance of one protein in the urine is less than or equal to that in serum, it was considered as serum leaked protein; If the relative abundance of one protein in the urine is greater than that in serum, it might be a urinary enriched protein, which could be considered as IgAN related protein. The relative abundance of TIMP1 and ICAM1 in the urine of IgAN was higher than those in the serum. These two proteins were enriched in the urine, indicating TIMP1 and ICAM1 were not simply leaked from the serum, but generated from the IgAN. The relative abundance of SERPINC1 and ADIPOQ in the urine of IgAN was lower than those in the serum, thus, serum-leakage could not be completely eliminated. (Additional file 4: Table S4).

In the glomerulonephropathy, the serum proteins might be leaked into the urine, and led to proteinuria. To further confirm whether the increase of 4 proteins is caused by serum-leakage or by IgAN, we compared the changes of four proteins in the urine of IgAN and other glomerulonephropathy (microalbuminuria DN). If one protein is up-regulated in both IgAN and microalbuminuria DN, it might be caused by serum-leakage; if one protein is only up-regulated in IgAN, it might be caused by IgAN itself. Guo et al. [6] compared the urinary N-glycoproteins in normal control, DN normalbuminuria, DN microalbuminuira and DN macroalbuminuria. ICAM1 and TIMP1 were not increased in the urine of microalbuminuria compared to the normalbuminuria, indicating these two proteins were not serum leaked proteins. While the SERPINC1 increase in the urine of microalbuminuria and macroalbuminuria [6]. ADIPOQ was O-glycoprotein, and was not identified in Guo’s study. In another study [35], ADIPOQ was up-regulated in the urine of DN (macroalbuminuria) compared to healthy control. These results indicated that SERPINC1 and ADIPOQ might be serum leaked proteins. However, the serum level of ADIPOQ also elevated in IgAN, the possible reason of the increase of ADIPOQ in urine needs further study (Additional file 4: Table S4).

In summary, the increase of ADIPOQ and SERPINC1 might originated from serum leakage, but TIMP1 and ICAM1 were not originated from serum leakage. Our study is a pilot research, the identification of biomarkers of IgAN were performed by comparison of IgAN patient and normal control. Though we compared the four biomarkers in the urine of IgAN with normal serum proteome to eliminate serum leaked proteins, it is difficult to completely exclude the serum leaked proteins. In our future study, to eliminate the influence of proteinuria, we will analyze the urinary proteomics in IgAN patients with different eGFR levels; meanwhile, we will further compare the urinary proteomics in IgAN with other related kidney diseases, which might identify the specific biomarkers of IgAN.

ICAM1 protein is a member of ligands for the leukocyte adhesion protein LFA-1 (integrin alpha-L/beta-2). The tubular and interstitial expression of ICAM1 can be a marker of tubulointerstitial disturbance in IgAN [30]. Interstitial ICAM1 could be an adverse predictor of disease progression. Several studies have reported that urinary ICAM1 is increased in acute renal allograft rejection [31], chronic kidney disease (CKD) [32] and lupus nephritis [33, 34]. This study provides the first demonstration that ICAM1 is overrepresented in the urine of IgAN patients. ICAM1 serves as a kidney injury biomarker and increases in varies renal pathologies [30]. However, we do not know whether the urinary ICAM1 levels showed differences or not among different renal pathologies. In our future study, we will compare the ICAM1 levels between IgAN and other kidney dieseases, and we would estimate whether ICAM1 could be used as a successful specific urinary biomarkers for IgAN or not.

In IgAN, the deposition of extracellular proteins is increased in the mesangium. MMPs are the zinc-dependent endopeptidases of the matrix metalloproteinase families that degrade extracellular membrane (ECM) proteins, and TIMPs are the endogenous inhibitors of MMPs. Thus, the degree of ECM is controlled by the release of MMPs and their inhibition by TIMPs. Previous studies have shown that TIMP1 is overexpressed in the kidney tissue of diabetic nephropathy rats [29]. TIMP1 is elevated in post-liver transplantation renal recovery from acute kidney injury after liver transplantation [28]. This study, which includes validation through a Western blot analysis, provides the first demonstration of the upregulation of TIMP1 in the urine of Uygur patients with IgA nephropathy. We speculated that TIMP1 serves as a positive regulator of ECMs and plays a role in the production and deposition of extracellular proteins in the mesangium during the course of IgA nephropathy.

**Fig. 5:**
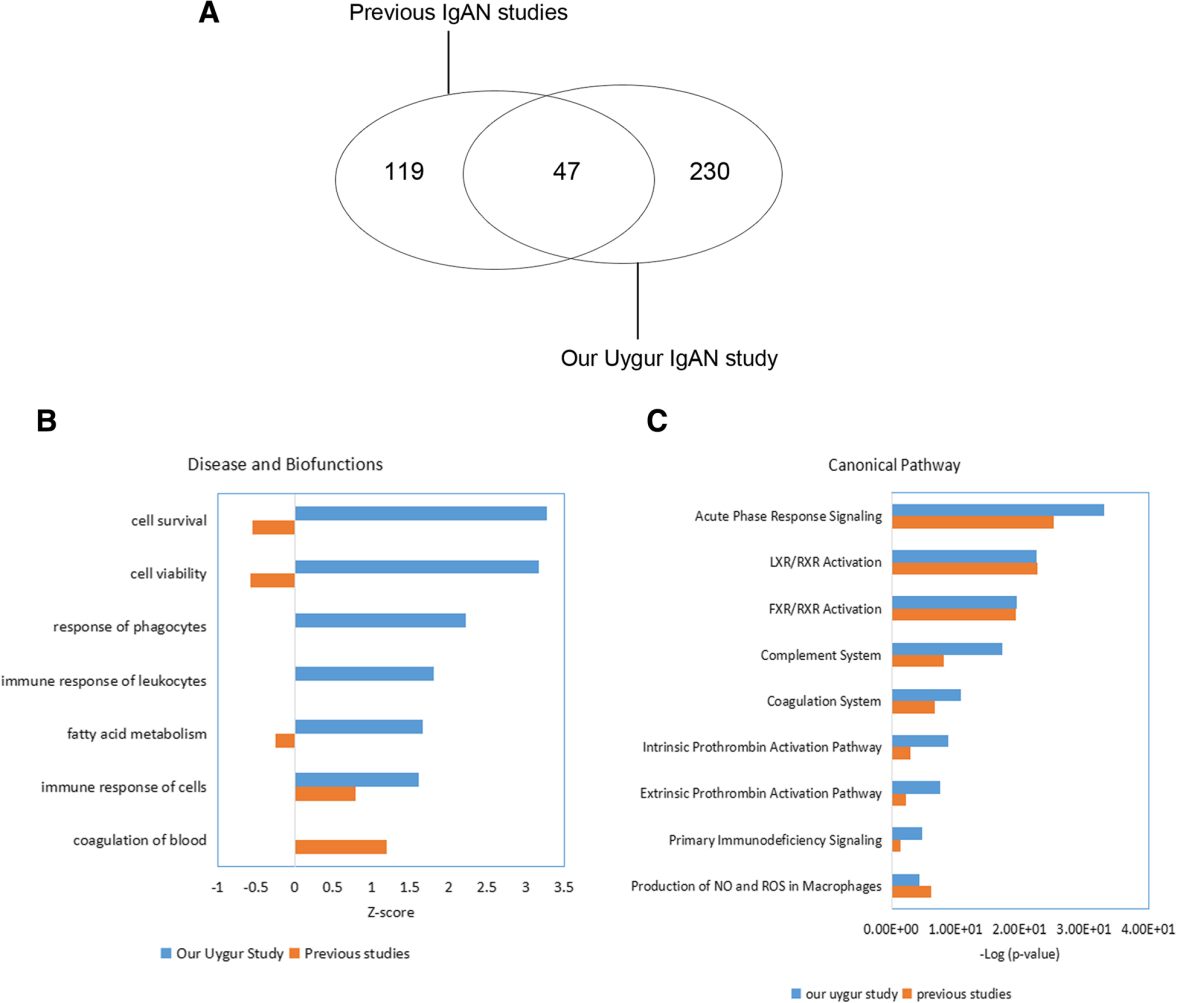
Comparison of differentially expressed proteins in IgAN identified in our Uygur study and previous studies. **a** Venn diagram of differentially expressed proteins in IgAN obtained in our Uygur study and previous studies. **b** Disease and biofunction analysis of differentially expressed proteins in IgAN obtained in our Uygur study and previous studies. Z-score > 2, significantly activated; Z-score < − 2, significantly inhibited. **c** Top enriched canonical pathways in IgAN identified in this Uygur study and previous studies. -Log(*p*-value) > 1.5, significantly enriched

To better understand the pathophysiological changes in IgAN, we compared the differentially expressed proteins in IgAN identified in previous studies with those obtained in our study. A total of 166 differential proteins were identified in previous publications [1, 7–11, 13, 39–43] (detailed data are shown in Additional file 5: Table S5). Only 47 of these overlapped with those found in our Uygur study (Fig. 5a). In addition, 36 of these 47 proteins showed consistent trends in this and previous studies.

An IPA analysis was performed to compare the differentially expressed proteins identified in previous studies and those identified in our Uygur study. The results of disease and functional analyses performed in previous studies and our study showed activation of the immune response in IgAN. More specifically, cell survival and viability, and phagocytosis were activated only in Uygur IgAN, as shown in our study (Fig. 5b). A pathway analysis revealed that the immune response and lipid metabolism-related pathway were enriched in IgAN in both previous studies and our Uygur study. This study found that the complement systems were more enriched in Uygur IgAN than previous studies (Fig. 5c). Therefore, our proteomic study reflects not only the common characteristics of IgAN that have been observed in previous studies but also special characteristics that are represented in Uygur IgAN but were not detected in previous studies. Because proteomic experiment of our study and previous studies are performed by different approaches and different laboratories, the differences results of IgAN urinary proteomics between our study and previous studies are still not very clear. It has been reported that IgAN shows marked variance between different ethnicities at the disease incidence and genome levels [14]. At the disease incidence level, IgAN is most common in Asians, is moderately prevalent in Europeans, and is rare in Africans [14]. At the genome level, Kiryluk et al. performed a GWAS to identify and confirm that five loci are significant contributors to the disease risk across this multi-ethnic cohort and used a genetic risk score to explain the worldwide patterns of disease prevalence [14]. Among the five loci, Chr.1q32 (CFHR3/R1 locus) encodes complement factor H (CFH), complement factor H-related protein 1 (CFHR1), CFHR2 and CFHR3 genes. Interestingly, CFH, and CFHR2 were all significantly upregulated in the urine of Uygur IgAN patients. We speculated that the genome variation in IgAN would affect the proteome and we speculated that there are differences in the IgAN proteome and the pathological mechanism between different ethnicities, and this hypothesis should be validated in a study that systematic compared between uygur population and other ethinities using the same approaches in the same laboratories.

## Conclusion

In this study, we analyzed the differential urinary proteome of Uygur IgAN. A function analysis revealed that the immune response, cell survival, and complement system were activated in Uygur IgAN. Four candidate biomarkers were validated by Western blot analysis, and all of these were found to be upregulated in Uygur IgAN patients. Our study found that some of the differential proteins might be results of proteinuria. To identify the specific IgAN biomarkers, the proteinuia originated differential proteins should be eliminated in the future studies. Our findings would be helpful for a future exploration of the pathological mechanism of Uygur IgAN and will be helpful for the development of better treatments for IgAN patients of Uygur ethnicity.

## Additional files

Additional file 1: **Table S1.** Clinical characteristics of Uygur normal controls and IgAN patients. (XLSX 13 kb)

Additional file 2: **Table S2.** Quantitative protein and peptide data. Quantitative values of peptides and proteins in the Uygur control and IgAN patients in runs 1 and 2 are shown. The sequence coverage of each protein in runs 1 and 2 are shown. (XLSX 421 kb)

Additional file 3: **Table S3.** Differentially expressed proteins in Uygur IgAN. (XLSX 19 kb)

Additional file 4: **Table S4.** Analysis the origin of the 4 candidate markers. (XLSX 10 kb)

Additional file 5: **Table S5.** Lists of differentially expressed proteins in IgAN obtained in previous studies. (XLS 66 kb)

## Abbreviations

ACN: Acetonitrile
Alb: Serum albumin
AUC: Area-under-the-curve
BMI: Body mass index
BUN: Blood urea nitrogen
DBP: Diastolic blood pressure
DTT: Dithiothreitol
ECM: Extracellular membrane
eGFR: Estimated glomerular filtration rate
FASP: Filter-aided sample preparation
FBG: Fasting blood glucose
FDR: False discovery rate
GWAS: Genome-wide association study
HDL: High-density lipoprotein
IAM: Iodoacetamide
IPA: Ingenuity Pathway Analysis
iTRAQ: Isobaric tags for relative and absolute quantitation
LDL: Low-density lipoprotein
SBP: Systolic blood pressure
SCr: Serum creatinine
TG: Triglycerides
UAER: Urinary albumin excretion rates
